# Divergent surveillance needs and resource allocation for COVID-19 and influenza: insights from a community-based syndromic surveillance study in Shanghai (2024–2025)

**DOI:** 10.3389/fpubh.2026.1777017

**Published:** 2026-03-03

**Authors:** Xiao Yu, Shiying Yuan, Huanyu Wu, Shenghua Mao, Sheng Lin, Xianjin Jiang, Xiaohuan Gong, Chenyan Jiang, Yaxu Zheng, Jian Chen

**Affiliations:** 1 Institute for Infectious Disease Control and Prevention, Shanghai Municipal Center for Disease Control and Prevention (Shanghai Academy of Preventive Medicine), Shanghai, China; 2 Institute for Surveillance and Early Warning, Shanghai Municipal Center for Disease Control and Prevention (Shanghai Academy of Preventive Medical Sciences), Shanghai, China

**Keywords:** community-based syndromic surveillance, COVID-19, healthcare-seeking, ILI, influenza, symptom profiles

## Abstract

**Introduction:**

The rapid evolution and symptom overlap of Coronavirus disease 2019 (COVID-19) and influenza challenge the effectiveness of current surveillance and healthcare resource planning. However, comparative evidence regarding their surveillance sensitivity and healthcare burden remains limited, particularly within concurrent community populations that capture the full spectrum of disease severity.

**Methods:**

To address this gap, data were derived from a community-based syndromic surveillance cohort in Shanghai, followed weekly between May 2024 and August 2025. We analyzed symptom profiles and illness duration, assessed the sensitivity of Influenza-Like Illness (ILI) definitions, and evaluated healthcare-seeking behaviors across both acute (0–14 days) and post-acute (>14 days) phases.

**Results:**

From May 2024 to August 2025, 382 COVID-19 and 175 influenza cases were identified. Compared with influenza, COVID-19 cases presented distinctively with upper respiratory symptoms (sore throat: 72.88% vs. 58.29%, runny or stuffy nose: 46.58% vs. 33.71%, loss of taste or smell: 3.84% vs. 0.57%; all *p* < 0.05), rather than fever (61.64% vs. 74.86%, *p* = 0.003). Consequently, standard ILI definitions failed to detect a significantly larger proportion of COVID-19 cases compared to influenza (China CDC criteria: 35.89% vs. 50.86%, *p* = 0.001; WHO criteria: 27.12% vs. 44.00%, *p* < 0.001). While illness duration was shorter for COVID-19 (6.66 ± 4.35 days vs. 8.25 ± 4.34 days, *p* < 0.05), influenza imposed a heavier healthcare burden, characterized by a two-fold increase in outpatient visits during the acute phase (OR = 2.12, 95% CI: 1.52–2.95) and sustained demand in the first 90 days of the post-acute phase (HR = 1.29, 95% CI: 1.03–1.61).

**Conclusion:**

COVID-19’s symptom profile limits ILI surveillance sensitivity, whereas influenza imposes a higher burden extending into the post-acute phase. These differences call for adapting surveillance strategies and healthcare resource allocation to these distinct pathogen profiles.

## 1 Introduction

Acute respiratory infections (ARIs) driven by severe acute respiratory syndrome coronavirus 2 (SARS-CoV-2) and influenza viruses remain significant contributors to global morbidity and mortality. Since late 2019, SARS-CoV-2 has spread globally, resulting in over 776 million reported cases (1), while seasonal influenza causes an estimated 3–5 million cases of severe illness and 290,000–650,000 deaths annually (2).

Following the 2009 H1N1 influenza pandemic, the World Health Organization (WHO) standardized the case definition for Influenza-Like Illness (ILI)—typically fever (≥38 °C) and cough—specifically to capture influenza activity (3). In response to the COVID-19 pandemic, surveillance systems worldwide have integrated SARS-CoV-2 testing into these existing influenza sentinel networks to maximize resource efficiency (4, 5). However, while ILI serves as a robust benchmark for influenza, its effectiveness in capturing current COVID-19 variants appears to be diminishing. This challenge intensified with the emergence of the Omicron subvariant BA.2.86 and its descendants, which arose in late 2023 carrying numerous spike mutations and rapidly achieved global predominance in early 2024 (6). Recent observations indicate that unlike ancestral strains, Omicron variants are increasingly characterized by upper respiratory symptoms (e.g., sore throat) rather than the systemic febrile response (7). Consequently, the sensitivity of standard fever-based definitions is increasingly being challenged when applied to these modern variants (8), raising concerns that relying strictly on ILI definitions may introduce a surveillance bias that obscures the true burden of COVID-19 compared to influenza.

Beyond surveillance, effective public health preparedness demands a precise understanding of healthcare resource utilization, encompassing illness duration and care-seeking behaviors (9). However, current evidence regarding the comparative healthcare burden of COVID-19 and influenza remains inconsistent. While large-scale hospital-based cohorts have demonstrated that COVID-19 generally carries a higher risk of post-acute multi-organ sequelae and mortality, notable distinctions emerge: the same comparative analyses highlight that influenza imposes a significantly higher burden specifically on the pulmonary system (10). Furthermore, in the acute phase, recent studies indicate that influenza generates heavier outpatient demand driven by a significantly higher prevalence of high-grade fever compared to Omicron variants (11). These distinct profiles suggest that the two viruses drive differential healthcare demands across the acute and post-acute phases. Therefore, clarifying these phase-specific utilization patterns is essential for optimizing resource allocation.

To address these knowledge gaps, the primary objective of this study was to directly compare the clinical profiles, surveillance sensitivity, and healthcare burden of COVID-19 and influenza under a unified framework. We analyzed data from a community-based, prospective syndromic surveillance cohort in Shanghai to achieve this. The novel contributions of this work are threefold. First, unlike hospital-based studies, our prospective community-based design minimizes selection bias and captures the full spectrum of disease severity, including mild cases. Second, we explicitly contrast symptom profiles to quantify the specific sensitivity gap of standard ILI definitions for current COVID-19 variants. Third, we extend the comparative analysis into the post-acute phase, providing critical evidence on long-term healthcare utilization differences. The remainder of the paper is structured as follows: we first detail the study design and data collection; then present the comparative results on symptom profiles and healthcare-seeking behaviors; and finally discuss the implications for surveillance policy and resource allocation.

## 2 Materials and methods

### 2.1 Study population

This study included 382 incident COVID-19 patients and 175 influenza patients from a community-based syndromic surveillance cohort. The cohort was launched in May 2024 across all 16 districts of Shanghai. Enrollment comprised 15,199 residents, sampled to match the age-sex distribution of the 7th National Census. Weekly follow-ups maintained an average of 14,935 respondents per cycle through a hybrid surveillance model. Participants were encouraged to self-report symptoms (including onset and resolution dates) and antigen results via the Shanghai Health Cloud mobile application. For those who did not self-report, trained family physicians conducted supplementary telephone follow-ups and recorded the missing data into the application. For participants who reported symptoms, family physicians tracked their healthcare-seeking behaviors and recovery status. Participants meeting the clinical criteria were eligible for specimen collection (via clinic visits or home sampling), with results returned via the cloud platform within 24 h. Enrollment numbers and losses to follow-up over time are provided in Supplementary Material 1.

Eligibility for specimen collection in this cohort required either (1) ≥1 acute systemic symptom (e.g., fever, chills, hypothermia, myalgia) plus ≥1 respiratory symptom (e.g., cough, sputum, sore/dry throat, nasal congestion/rhinorrhea, anosmia, ageusia, dyspnea, chest pain, tachypnea, difficulty breathing), or (2) ≥2 respiratory symptoms. Respiratory specimens, either throat swabs or sputum, were collected for pathogen detection using targeted next-generation sequencing (tNGS); genotyping and variant identification were based exclusively on tNGS results (12).

COVID-19 and influenza cases were ascertained from three complementary sources within the cohort: Pathogen detection using tNGS; or Self-reported positive antigen test results; or hospital-based clinical diagnoses.

### 2.2 Data collection

Symptom follow-up data and pathogen detection results were managed on the Shanghai Health Cloud, which also integrated demographic information, vaccination history, and medical consultation records for real-time surveillance.

### 2.3 Definitions

The WHO definition of ILI includes symptom onset within the past 10 days, with both “fever (≥38 °C)” and “cough” (13). The China CDC definition of ILI requires “fever (≥38 °C)” along with at least one of the following symptoms: “cough” or “sore throat” (14).

### 2.4 Statistical analysis

Categorical variables were compared using *χ*^2^ or Fisher’s exact tests, and continuous variables using Student’s *t*-tests. Healthcare-seeking behaviors were evaluated in two distinct phases: (i) the acute phase (0–14 days from symptom onset), analyzed using multivariable logistic regression; and (ii) the post-acute phase (defined as >14 days after symptom onset), analyzed using Cox proportional hazards models to assess the time to the first all-cause outpatient visit. For the Cox analysis, the post-acute follow-up was partitioned into two intervals (0–90 days and 91–180 days from the start of the post-acute phase), and a disease-by-time interaction term (COVID-19 vs. influenza) was included to test for time-varying effects. Statistical significance was defined as a two-sided *p* < 0.05. All analyses were conducted using Python (version 3.11.7; pandas, numpy, scipy, statsmodels) and R (version 4.5.0; R Foundation for Statistical Computing).

## 3 Results

### 3.1 Demographics and temporal patterns

From May 9, 2024, to August 13, 2025, 382 COVID-19 cases (266 tNGS-confirmed) and 175 influenza cases (138 tNGS-confirmed) were identified. Baseline characteristics were similar between COVID-19 and influenza groups for sex, comorbidities, BMI, and prior COVID-19 or influenza vaccination. However, age distribution differed, with a higher proportion of individuals aged 0–14 years in the influenza group (Table 1).

**Table 1:** Patient characteristics of COVID-19 and influenza cases.

Variable	COVID-19 (N, %)				Influenza (N, %)		*χ* ^2^	*p*
	tNGS detected (*N* = 266)	Antigen or hospital diagnosed (*N* = 116)	All (*N* = 382)	tNGS detected (*N* = 137)	Antigen or hospital diagnosed (*N* = 38)	All (*N* = 175)		
Sex							0.45	0.5
Male	94 (35.34)	48 (41.38)	142 (37.17)	59 (43.07)	12 (31.58)	71 (40.57)		
Female	172 (64.66)	68 (58.62)	240 (62.83)	78 (56.93)	26 (68.42)	104 (59.43)		
Age group							15.31	<0.01
0–14 years	24 (9.02)	11 (9.48)	35 (9.16)	20 (14.6)	17(44.74)	37 (21.14)		
15–59 years	219 (82.33)	83 (71.55)	302 (79.06)	101 (73.72)	19 (50)	120 (68.57)		
≥60 years	23 (8.65)	22 (18.97)	45 (11.78)	16 (11.68)	2 (5.26)	18 (10.29)		
Comorbidity							0.34	0.56
No	232 (87.22)	91 (78.45)	323 (84.55)	119 (86.86)	33 (86.84)	152 (86.86)		
Yes	34 (12.78)	25 (21.55)	59 (15.45)	18 (13.14)	5 (13.16)	23 (13.14)		
BMI categories*							3.45	0.33
Normal	143 (53.76)	57 (49.14)	200 (52.35)	70 (51.09)	19 (50)	89 (50.85)		
Underweight	95 (35.71)	39 (33.62)	134 (35.08)	48 (35.04)	13 (34.21)	54 (30.86)		
Overweight/obesity	25 (9.4)	19 (16.38)	44 (11.52)	16 (11.68)	6 (15.79)	29 (16.57)		
Missing	3 (1.13)	1 (0.86)	4 (1.05)	3 (2.19)	0 (0)	3 (1.72)		
COVID-19 vaccine (1 year prior onset)							1.93	0.10^†^
No	260 (97.74)	115 (99.14)	375 (98.16)	137 (100)	38 (100)	175 (100)		
Yes	6 (2.26)	1 (0.86)	7 (1.84)	0 (0)	0 (0)	0 (0)		
Flu vaccine (1 year prior onset)							1.13	0.29
No	203 (76.32)	101 (87.07)	305 (79.79)	106 (77.37)	26 (68.42)	132 (75.43)		
Yes	63 (23.68)	15 (12.93)	77 (20.21)	31 (22.63)	12 (31.58)	43 (24.57)		

*BMI categories: BMI < 18.5 kg/m^2^ is defined as underweight, 18.5 kg/m^2^ ≤ BMI < 24.0 kg/m^2^ is defined as normal weight. BMI ≥ 24.0 kg/m^2^ is defined as overweight/obesity. ^†^Fisher’s exact test was used for COVID-19 vaccination due to a zero cell.

**Figure 1:**
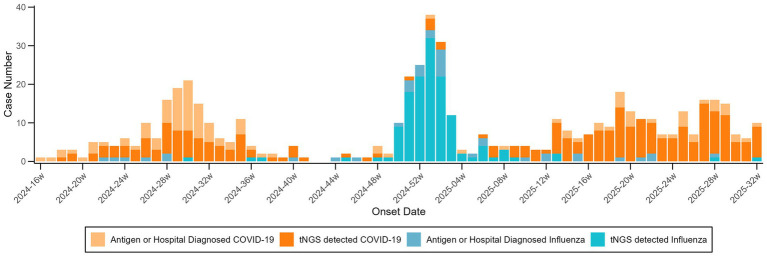
Onset time of COVID-19 and influenza cases during the study period. Time series of weekly onset counts for COVID-19 and influenza among cohort participants from May 9, 2024 through August 13, 2025.

COVID-19 activity clustered in epidemiological weeks 16–40 of 2024 and 13–32 of 2025, whereas influenza peaked from week 48 of 2024 to week 8 of 2025, showing a sharper peak but shorter duration (Figure 1).

Among tNGS-confirmed COVID-19 cases (*n* = 266), the predominant circulating strains were SARS-CoV-2 Omicron JN.1 (50.00%) and Omicron BA.2.86 (42.86%), with a small proportion untyped (7.14%). For influenza (*n* = 138), the majority of cases were caused by influenza A (H1N1) pdm09 (89.05%), followed by untyped IAV (5.84%), H3N2 (1.46%) and influenza C virus (3.65%).

### 3.2 Symptom profile and ILI capture

**Figure 2:**
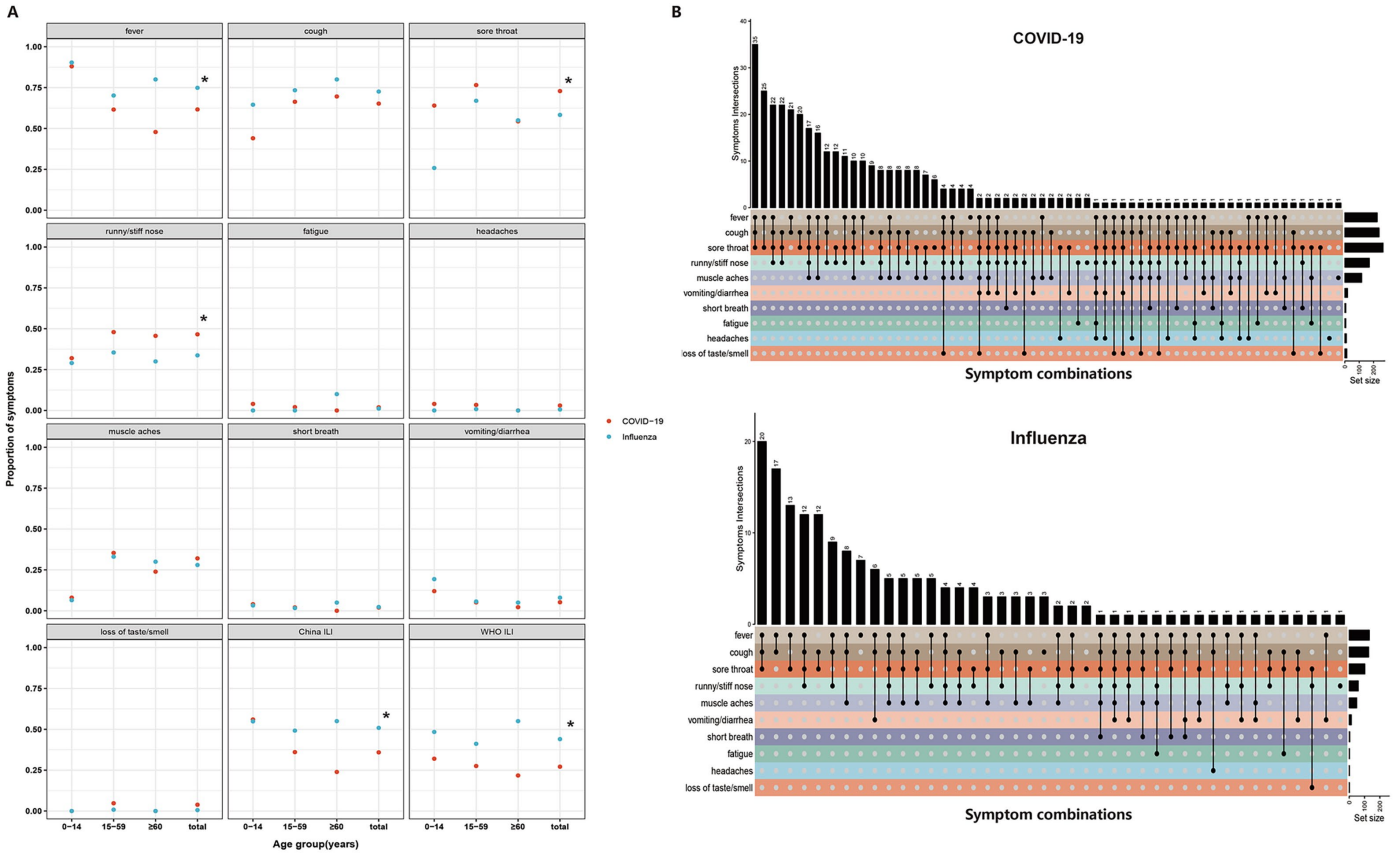
Frequency of symptoms and symptom combinations in COVID-19 and influenza cases. **(A)** Frequency of individual symptoms and proportions of cases meeting the China ILI and WHO ILI definitions among COVID-19 and influenza cases; “*” means *p* < 0.05. **(B)** Symptom combinations observed in COVID-19 and influenza cases. China ILI is defined as fever (>38 °C) with either cough or sore throat. WHO ILI is defined as fever (>38 °C) with cough. ILI, influenza-like illness.

Figure 2A summarizes the symptom frequencies for COVID-19 and influenza cases. Distinct clinical profiles emerged between the two groups. COVID-19 cases were significantly more likely to report upper respiratory symptoms, including sore throat (72.88% vs. 58.29%, *p* < 0.001), runny or stuffy nose (46.58% vs. 33.71%, *p* = 0.006), and loss of taste or smell (3.84% vs. 0.57%, *p* = 0.046). In contrast, influenza cases showed more fever (74.86% vs. 61.64%, *p* = 0.003). No significant differences were observed in the prevalence of cough (COVID-19: 65.21% vs. Influenza: 72.57%, *p* = 0.107), muscle aches (COVID-19: 32.05% vs. Influenza: 28.00%, *p* = 0.392), fatigue (COVID-19: 1.92% vs. Influenza: 1.14%, *p* = 0.725), headaches (COVID-19: 3.01% vs. Influenza: 0.57%, *p* = 0.115), or vomiting/diarrhea (COVID-19: 5.21% vs. Influenza: 8.00%, *p* = 0.282) between the two groups.

Reflecting these symptom disparities, the sensitivity of ILI definitions for detecting COVID-19 was significantly lower compared to influenza. Under the China CDC criteria, sensitivity was 35.89% for COVID-19 versus 50.86% for influenza (*p* = 0.001). This gap widened under the WHO criteria (27.12% vs. 44.00%, *p* < 0.001). Subgroup analysis indicated that this reduced sensitivity was evident across all age groups (Figure 2A).

Analysis of co-occurring symptoms (Figure 2B) further highlighted these divergences. While fever, sore throat, cough, and rhinitis were common in both cohorts, their combinatorial patterns differed. Notably, the combination of cough, sore throat, and runny/stuffy nose ranked as the third most frequent combination in COVID-19 cases but was rare in influenza cases, reinforcing the distinct upper-respiratory presentation of current SARS-CoV-2 variants.

### 3.3 Illness duration and healthcare-seeking behavior

The average illness duration was significantly shorter in COVID-19 cases (6.66 ± 4.35 days) compared to influenza cases (8.25 ± 4.34 days; *t* = −3.96, *p* < 0.05). Outpatient visit rates were 13.87% (95% CI, 10.41%–17.34%) for COVID-19 cases and 33.71% (95% CI, 26.71%–40.72%) for influenza cases (*χ*^2^ = 28.19, *p* < 0.001). Hospitalizations were rare (1 case for COVID-19; 2 cases for influenza). As shown in Table 2, after adjusting for multiple factors, influenza infection was associated with more than a two-fold increase in the likelihood of healthcare-seeking compared to SARS-CoV-2 infection (Odds ratio [OR] = 2.12, 95% CI, 1.52–2.95, *p* < 0.001). Age had a clear effect: individuals aged 15–59 years were less likely to seek care than those aged 0–14 years (OR = 0.61, 95% CI, 0.38–1.00, *p* = 0.05), while cases aged ≥60 years did not significantly differ from the youngest group (OR = 1.04, 95% CI, 0.54–1.99, *p* = 0.91). Sex, comorbidities, and BMI did not show significant associations.

**Table 2:** Multivariable logistic regression of factors associated with outpatient visits within 14 days after symptom onset of COVID-19 and influenza cases.

Variable	*β* (coef)	SE	OR (95% CI)	*p*
Intercept	−1.49	0.28	0.23 (0.13–0.40)	<0.001
Disease				
COVID-19	Ref	Ref	Ref	–
Influenza	0.75	0.17	2.12 (1.52–2.95)	<0.001
Sex				
Male	Ref	Ref	Ref	–
Female	−0.15	0.17	0.86 (0.62–1.20)	0.38
Age group				
0–14 years	Ref	Ref	Ref	–
15–59 years	−0.49	0.25	0.61 (0.38–1.00)	0.05
≥60 years	0.04	0.33	1.04 (0.54–1.99)	0.91
Comorbidity				
No	Ref	Ref	Ref	–
Yes	0.25	0.25	1.29 (0.79–2.09)	0.31
BMI categories^*^				
Normal	Ref	Ref	Ref	–
Underweight	−0.15	0.2	0.86 (0.58–1.27)	0.46
Overweight/obesity	0.2	0.24	1.22 (0.76–1.96)	0.41
COVID-19 vaccine (1 year prior onset)				
No	Ref	Ref	Ref	–
Yes	−35.25	NE^†^	NE^†^	0.99
Flu vaccine (1 year prior onset)				
No	Ref	Ref	Ref	–
Yes	−0.26	0.22	0.77 (0.50–1.19)	0.24

*BMI categories: BMI < 18.5 kg/m^2^ is defined as underweight, 18.5 kg/m^2^ ≤ BMI < 24.0 kg/m^2^ is defined as normal weight. BMI ≥ 24.0 kg/m^2^ is defined as overweight/obesity. ^†^Not estimable due to quasi-complete separation.

When examining the risk of all-cause outpatient visits during the post-acute phase (Table 3), significant temporal differences were observed between the two disease groups. During the early post-acute interval (0–90 days), influenza cases demonstrated a significantly higher hazard of healthcare-seeking compared with COVID-19 cases (HR = 1.29; 95% CI: 1.03–1.61; *p* = 0.03). However, this disparity diminished over time; in the subsequent 91–180-day interval, the difference between the groups was no longer statistically significant (HR = 1.28; 95% CI: 0.37–4.46; *p* = 0.70). Independent of viral etiology, older age and the presence of comorbidities were consistently associated with increased healthcare utilization, whereas BMI and vaccination history did not show significant associations.

**Table 3:** Multivariable Cox regression analysis of time to first all-cause outpatient visit during the post-acute phase following COVID-19 and influenza infection.

Variable	*β* (coef)	SE	HR (95% CI)	*p*
Disease × time				
Influenza vs. COVID-19 (0–90 d)	0.25	0.11	1.29 (1.03–1.61)	0.03
Influenza vs. COVID-19 (91–180 d)	0.25	0.64	1.28 (0.37–4.46)	0.70
Sex				
Male	Ref	Ref	Ref	
Female	0.38	0.14	1.47 (1.11–1.93)	0.01
Age group				
0–14 y	Ref	Ref	Ref	
15–59 y	1.10	0.23	3.01 (1.91–4.77)	<0.001
≥60 y	1.56	0.29	4.74 (2.69–8.36)	<0.001
Comorbidity				
No	Ref	Ref	Ref	
Yes	0.63	0.21	1.87 (1.23–2.84)	0.003
BMI categories^*^				
Normal	Ref	Ref	Ref	
Underweight	−0.15	0.15	0.86 (0.64–1.16)	0.20
Overweight/obesity	0.24	0.21	1.27 (0.85–1.89)	0.25
COVID-19 vaccine (1 year prior onset)				
No	Ref	Ref	Ref	
Yes	0.4	0.43	1.5 (0.64–3.50)	0.35
Flu vaccine (1 year prior onset)				
No	Ref	Ref	Ref	
Yes	0.17	0.18	1.19 (0.83–1.70)	0.35

*BMI categories: underweight (<18.5 kg/m^2^), normal weight (18.5–23.9 kg/m^2^), and overweight/obesity (≥24.0 kg/m^2^). Model specification: hazard ratios (HRs) were estimated using a Cox proportional hazards model with a time-by-disease interaction term to allow for varying risks across time intervals. The intervals “0–90 days” and “91–180 days” refer to the post-acute phase (starting 14 days after symptom onset). HRs represent the risk of outpatient visits for influenza compared to COVID-19 within each specific period. Adjustments: models were adjusted for age, sex, comorbidities, BMI, and vaccination history (both COVID-19 and influenza). CI, confidence interval; HR, hazard ratio.

## 4 Discussion

In this community-based prospective cohort study, we identified a distinct divergence in the clinical profiles, surveillance sensitivity, and healthcare-seeking behaviors of COVID-19 and influenza. While influenza maintained a classic febrile presentation driving high medical demand, Omicron-era COVID-19 manifested as a milder, often afebrile upper respiratory illness that frequently evaded detection under standard ILI surveillance criteria.

Consistent with prior evidence regarding the Omicron variant (7), COVID-19 cases in our cohort presented with less fever and more sore throat. This phenotypic divergence is likely driven by distinct viral tropisms and altered cell entry mechanisms. Unlike influenza viruses, which typically induce robust systemic inflammation, recent virological studies indicate that Omicron variants have evolved a reduced reliance on the cell-surface protease TMPRSS2 for entry, favoring the endosomal pathway instead (15). This evolutionary shift limits viral replication in the lung parenchyma while enhancing efficiency in the ACE2-rich oropharyngeal epithelium, thereby mechanistically explaining the predominance of upper respiratory symptoms such as sore throat over systemic severity (16, 17). This clinical profile stands in stark contrast to the high-grade fever that continues to characterize seasonal influenza A infections (11). Consequently, this poses a fundamental challenge to traditional surveillance. Our findings suggest that strictly fever-based ILI criteria may underestimate COVID-19 activity. To enhance surveillance sensitivity, adopting a broader ARI framework warrants consideration. This approach aligns with the integrated surveillance strategy employed by the US CDC, which utilizes broad syndromic metrics to capture the aggregate burden of co-circulating respiratory viruses (18). Although including afebrile symptoms inevitably lowers clinical specificity (19), ARI serves as a sensitive “screening net” for sentinel surveillance. In this model, high sensitivity ensures the capture of mild COVID-19 cases, while specificity is subsequently confirmed by laboratory testing. Thus, monitoring ARI trends alongside virological positivity rates is essential for accurate epidemic assessment during periods of co-circulation.

Healthcare utilization patterns mirrored symptomatic differences. COVID-19 cases exhibited significantly lower short-term healthcare-seeking rates (13.87% vs. 33.71%) and shorter illness duration (6.66 vs. 8.25 days). While pre-Omicron variants were associated with greater severity (20), our findings align with recent evidence showing shorter hospital stays for Omicron (6.20 days) than influenza (8.30 days) (21). In the post-acute phase, influenza cases retained a higher outpatient burden over 90 days, aligning with findings from Taiwan showing lower post-acute utilization for COVID-19 (22). This sustained demand challenges the perception that influenza recovery is immediate. While Long COVID rightly garners attention for its multi-organ sequelae, recent large-scale cohorts clarify that for the pulmonary system specifically, the long-term risks associated with seasonal influenza can actually exceed those of Omicron (10). Mechanistically, influenza’s higher propensity for secondary bacterial co-infections likely compounds this persistent burden, driving both prolonged symptomatic recovery and a sustained reliance on outpatient care beyond the acute phase (23).

This divergence in clinical severity likely shapes transmission dynamics. In Shanghai, COVID-19 activity persisted across three seasons, contrasting with the sharper, shorter winter peak of influenza. The more frequent mild or afebrile presentation of Omicron-era COVID-19 may reduce the effectiveness of symptom-triggered care seeking and isolation, allowing community activity to persist and remain harder to detect (24, 25). Conversely, influenza more often causes acute febrile illness that physically incapacitates patients (26); however, due to its strong seasonality, it tends to concentrate healthcare demand into a compressed, high-intensity period (27). This creates a distinct public health paradox: COVID-19 requires a wide, sensitive net to detect invisible transmission, whereas influenza demands a robust, resilient buffer to absorb visible clinical surges.

A key strength of this study is its prospective, community-based design, which captures mild, non-attending cases often missed by hospital-based surveillance. Furthermore, linkage with the Shanghai Health Cloud allowed for the integration of longitudinal follow-up with real-world healthcare utilization records. However, limitations exist. First, self-reported symptoms are subject to recall bias, though weekly follow-ups aimed to mitigate this. Second, voluntary testing may introduce selection bias; yet, such bias is likely non-differential and unlikely to alter comparative conclusions. Third, as our findings reflect specific variants (Omicron BA.2.86 and A (H1N1)pdm09), continuous re-evaluation is warranted given the rapid evolution of respiratory viruses.

In conclusion, this prospective community-based study characterized the distinct burden profiles of Omicron-era COVID-19 and seasonal influenza. Our findings demonstrated that while influenza maintained a classic febrile presentation, Omicron variants manifested primarily as mild upper respiratory infections that frequently evaded detection under standard ILI surveillance criteria. Furthermore, longitudinal follow-up revealed that influenza imposed a more significant burden on outpatient resources during the acute and early post-acute phases compared to the milder clinical trajectory of Omicron cases.

Looking forward, effective post-pandemic management requires calibrating public health strategies to these distinct viral profiles. We recommend that surveillance systems transition from fever-based ILI indicators to broader ARI metrics to accurately capture the burden of current and future SARS-CoV-2 variants. Additionally, resource allocation planning must account for the divergent demands of these pathogens: maintaining high-sensitivity monitoring to track community transmission of SARS-CoV-2, while ensuring resilient clinical capacity to manage the intense healthcare surges driven by seasonal influenza.

## Data Availability

The datasets presented in this article are not readily available because they contain information that could compromise the privacy of participants and are subject to the data management regulations of the Ethics Committee. Requests to access the datasets should be directed to the corresponding author.
