# Supplementary material for: Divergent surveillance needs and resource allocation for COVID-19 and influenza: insights from a community-based syndromic surveillance study in Shanghai (2024–2025)
Source: Front Public Health. 2026 Mar 3;14:1777017. doi: 10.3389/fpubh.2026.1777017 (PMC12992253; doi:10.3389/fpubh.2026.1777017)
Supplement: Supplementary file 1 [file Supplementary_file_1.docx]

## Supplementary Figures


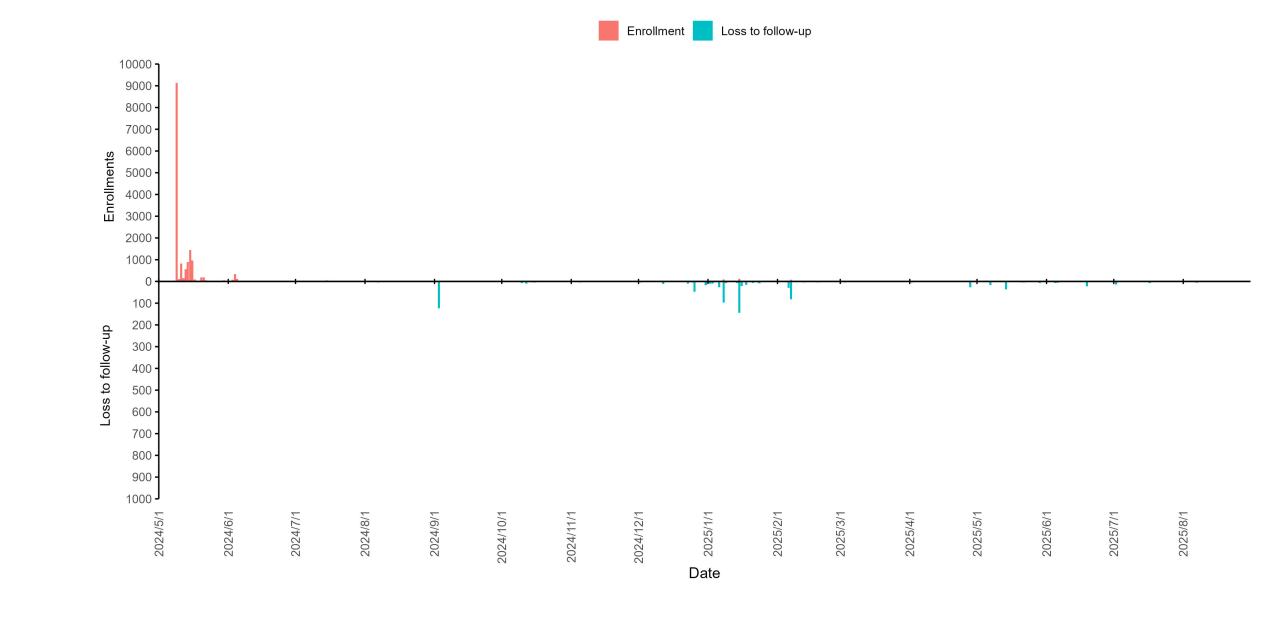


**Supplementary Figure 1.** Enrollments and losses to follow-up in the community syndromic surveillance cohort during the study period. Bars above the x-axis show the number of new participants enrolled on each date, and bars below the x-axis show the number of participants lost to follow-up.
